# Effect of Oropharyngeal Administration of Colostrum in Premature Newborns ≤32 Weeks of Gestation on the Immune Response and Neonatal Morbidity: A Double-Blind Randomized Clinical Trial

**DOI:** 10.3389/fped.2022.891491

**Published:** 2022-07-08

**Authors:** Silvia Romero-Maldonado, Diana Mercedes Soriano-Becerril, Perla Karina García-May, Enrique Reyes-Muñoz, Eudoxia Georgina Muñoz-Ortíz, Sandra Carrera-Muiños, Martha Lucía Granados-Cepeda, Jorge Arturo Cardona-Pérez, Elsa Castro-Millán, Enrique Segura-Cervantes, Guillermo Ceballos, Araceli Montoya-Estrada

**Affiliations:** ^1^Coordination of the Human Milk Bank, National Institute of Perinatology, Mexico City, Mexico; ^2^Immunology Department, National Institute of Perinatology, Mexico City, Mexico; ^3^Neonatal Intensive Care Unit, Hospital Adolfo López Mateos of the ISSSTE, Mexico City, Mexico; ^4^Coordination of Gynecological and Perinatal Endocrinology, National Institute of Perinatology, Mexico City, Mexico; ^5^Newborn Intensive Care Unit, Rio University Hospital, Cuenca, Ecuador; ^6^Newborn Intensive Care, National Institute of Perinatology, Mexico City, Mexico; ^7^Immediate Newborn Care Unit, Institute of Perinatology, Mexico City, Mexico; ^8^General Direction, National Institute of Perinatology, Mexico City, Mexico; ^9^Infectious Diseases and Epidemiology Unit, National Institute of Perinatology, Mexico City, Mexico; ^10^Postgraduate and Research Section, Higher School of Medicine, National Polytechnic Institute, Mexico City, Mexico

**Keywords:** colostrum, immunoglobulins, mortality of premature, premature, neonatal sepsis, tolerance to the enteral route

## Abstract

**Introduction:**

The mother's colostrum carries immunological components, such as cytokines and immunoglobulins (Igs), derived from the maternal circulation with bacteriostatic properties.

**Objective:**

The objective of this study was to evaluate the effect of oropharyngeal administration of colostrum (OPAC) vs. placebo in the first 4 days of life in premature newborns ≤32 weeks of gestation on serum Ig concentration, neonatal morbidity, and total days of hospitalization.

**Hypothesis:**

The OPAC increases serum Igs and decreases morbidity and total days of hospitalization.

**Materials and Methods:**

A double-blind randomized controlled trial was carried out. Participants were randomly assigned to one of the two groups, namely, group 1: placebo (P) (*n* = 50) and group 2: colostrum (C) (*n* = 46). A blood sample was obtained at baseline and 7 and 28 days of life to quantify immunoglobulin G (IgG), immunoglobulin A (IgA), and IgM. Results: The C group showed an increase in serum IgA on day 28 expressed as median and [interquartile range]; C: 25 [12–35] vs. P: 11 [8–18], *p* < 0.001. There were no significant differences in neonatal morbidity. Newborns in the colostrum group showed the completed enteral feeding earlier (days), C: 13.9 ± 7 vs. P: 17.4 ± 8.4, *p* < 0.04; they reached the birth weight earlier, C: 10.9 ± 2.8 vs. P: 12.9 ± 4, *p* < 0.01, and had less days of hospitalization, C: 60.2 ± 33.8 vs. P: 77.2 ± 47.3, *p* < 0.04. Neonatal mortality was lower in the colostrum grou*p* than the placebo group 0% vs. 12%, respectively, without a statistical difference (*p* = 0.06).

**Conclusion:**

In premature newborns ≤32 weeks of gestation, the OPAC within 4 days after birth increases serum IgA concentration at day 28 compared to placebo. Similarly, OPAC decreased the days to complete enteral feeding and reach the birth weight and total days of hospitalization.

**Clinical Trial Registration:**

[https://clinicaltrials.gov/ct2/show/NCT03578341], identifier: [NCT03578341].

## Introduction

Preterm birth is a live birth that occurs before 37 completed weeks of gestation. Approximately 15 million babies are born preterm annually worldwide, indicating a global preterm birth rate of about 11% (1, 2). Low-weight and extremely low-weight newborns are at risk of presenting complications, of which the most frequent are nosocomial sepsis (20–54% in those under 1,000 g), with a high mortality rate of close to 18% (3); necrotizing enterocolitis (NEC) with frequencies of 2–7% in premature newborns ≤32 weeks of gestation, and 5–22% in those <1,000 g (4); other frequent complications include intraventricular hemorrhage, bronchopulmonary dysplasia (BPD), and immune alterations related to gestational age (3).

Active transport of immunoglobulin G (IgG) across the placental syncytiotrophoblast is dependent on maternal placental IgG concentrations beginning in the second trimester of pregnancy. Transport occurs linearly as pregnancy progresses, and the most significant amount is transferred in the last trimester, reaching 50% of maternal concentrations between gestational weeks 28 and 32. Premature newborns have significantly lower IgG concentrations than preterm newborns (5).

Immunoglobulin A (IgA) inhibits the adherence of pathogens in the respiratory tract and the epithelium of the intestinal mucosa, functioning as a barrier (6). The number of immunoglobulins (Igs) found in colostrum on days 1–3 is IgG: 80–50 mg, IgM: 120–40 mg, IgA: 11,000–2,000 mg, and 1 to 3 g in mature milk, which means that the breastfed newborn receives passive immunity against viral and bacterial infections (7). These Igs are decreasing, and transitional milk contains less than colostrum; however, they remain effective during the duration of lactation.

The current recommendation to start feeding, including premature newborns, is breast milk. Colostrum is produced during the first 5 days postpartum and contains many immunological factors, growth factors, cytokines, leukocytes, and large amounts of nutrients (8). When administered in the oropharynx, it activates oral and intestinal immunity, protecting premature infants from various inflammatory diseases, such as sepsis and NEC (9).

Colostrum also contains oligosaccharides that can be absorbed through the oral mucosa with systemic effects to protect against the development of NEC (10).

Recently, several studies have explored the effect of oropharyngeal administration of colostrum (OPAC) on infectious processes, and Rodríguez N et al. (11) administered 0.2 ml colostrum in nine patients in the oropharyngeal mucosa every 2 h for 48 h, starting at 48 h of life and compared with six patients who were administered placebo; they found no difference between the groups in lactoferrin, IgA, and IL-10 levels in tracheal aspirates and urine. However, the group with colostrum completed the enteral route earlier, 14.3 ± 5.7 vs. 24.2 ± 8.7 days (*p* < 0.032).

Other small studies analyzing the effects of the administration of colostrum for 48 h reported the results that are still inconclusive (12). Abd-Elgawad et al. (13) reported that infants receiving oral colostrum had a significantly shorter Neonatal Intensive Care Unit (NICU) length of stay compared with infants with regular feeding. This effect is probably related to early achievement of discharge criteria in terms of being off oxygen therapy, on full oral feeding, and reaching the required weight, even if there are two meta-analyses where it is reported no effect of oral colostrum therapy on the length of NICU stay.

A systematic review that includes six randomized clinical trials showed that the days to complete enteral feeds were reduced in the early oropharyngeal colostrum group compared to placebo with mean differences of−2.58 days (95% CI−4.01 to−1.14; six studies, 335 infants; *P* = 0.0004; I^2^ = 28%; very low-quality evidence) (14). However, researchers found no significant differences between early oropharyngeal colostrum and control for incidence of NEC, late-onset infection, death before hospital discharge, and length of hospital stay. The authors concluded that large, well-designed trials would be required to evaluate more precisely and reliably the effects of oropharyngeal colostrum on important outcomes for preterm infants (14).

Given the lack of conclusive information, the primary aim of this study was to compare the effect of OPAC vs. placebo in the first 4 days of life in premature newborns≤32 weeks of gestation on serum Ig concentration, neonatal morbidity, and total days of hospitalization.

## Materials and Methods

### Study Design

A double-blind randomized clinical trial was carried out. The study was performed according to the principles of the Declaration of Helsinki and was approved by the Internal Review Board of the National Institute of Perinatology “Isidro Espinosa de los Reyes” and registered with a number (546) 212250-2320-10305-01-16. This clinical trial was registered with a number NTC03578341. Informed consent was signed by the mother of each participant.

### Description of the Study Subjects

Premature newborns <32 weeks of gestation were included from 1 June 2018 to September 2019. Before administering the first dose of colostrum, 1 ml of blood was drawn for the serum quantification of Igs; the procedure was repeated at 7 and 28 days. Premature newborns whose mothers were carriers of HIV, hepatitis B and C, cytomegalovirus, newborns with major congenital defects, hemolytic syndromes, early-onset sepsis, or who received transfusions in the first 24 h of life were not included in the study.

### Procedure

Participants were randomly assigned to one of the two groups: group 1: placebo (P) and group 2: colostrum (C), using a random number table. Both interventions (colostrum and placebo) were presented in the same packaging and appearance. The presentation was a sterile and 1-ml graduated plastic syringe that was covered with aluminum foil to cover the content. All mothers were asked to perform the expression manually or with a manual breast pump, regardless of their assigned group. The colostrum and placebo were prepared in the Human Milk Bank to perform the dosage of 0.3 ml of colostrum and 0.3 ml of double-distilled water for the placebo. Colostrum or placebo administration was started within 24 h after birth, every 4 h, for 4 days. Mothers extracted colostrum, twice a day, regardless of the group to which the newborns were assigned. The mothers of participants, research team, doctors, and the assistant nurse were blinded to the allocation group untilthe final analysis. The data were collected by two clinical doctors, who were blinded and did not take participation in the management of the patients.

The assistant nurse administered the colostrum or placebo drop by drop to the oropharyngeal mucosa of the newborn. During the administration, the nurse places the tip of the syringe in the child's mouth, along with the tissue of the right and left buccal mucosa, administering 0.15 ml (approximately three drops) of colostrum in each cheek of the mouth during a period at least 1 min with the tip directed toward the posterior part of the oropharynx; therefore, a total of 0.3 ml was administered per treatment session.

The institutional policy for feeding children under 1,500 g begins with the concept of aggressive nutrition; all premature infants receive solutions containing 2.8 to 3 g/kg/day of proteins, 4 mg/kg/min of glucose, and calcium gluconate; after 24 h, total parenteral nutrition is started at 3 g/kg/day of proteins, 2–3 g/kg/day of lipids, 4 mg/kg/min of carbohydrates to a total of 12.5 mg/kg/min (17.28 g/kg/day). The enteral nutrition begins with 12.5 ml/kg/day and is divided into eight intakes; depending on tolerance, it increases from 12.5 to 25 ml/kg/day with human milk or pasteurized donated milk up to 150 ml/kg/day; the total parenteral nutrition is gradually decreased and withdrawn upon reaching the volume mentioned. It is essential to mention that INPer has a Human Milk Bank and 100% of patients ≤32 weeks of gestation start with milk from their mother (40% homologous) or with pasteurized donated human milk (60%); however, for the next 15 days, premature newborns are fed 50/50% milk from their mother and with pasteurized donated human milk. After the first 15 days, once the newborns completed the enteral route, they continued to be fed with homologous and/or fortified pasteurized human milk (an acidified liquid fortifier is used, adding 4 vials of 5 ml of the fortifier to 100 ml of human milk). In addition, according to the tolerance of the enteral route, it is increased by 10 ml/kg/day until reaching 180 ml/kg/day. Newborns receive a multivitamin from the first week, which is continued daily during their hospital stay (vitamin A: 1,500 IU/ml, vitamin C: 30 mg/ml, and vitamin D: 400 IU/ml). Fifteen days after birth, they start with iron and folic acid, in a presentation of 600 mg/10 mg/100 ml (dosed at 2–4 mg/kg/day of iron), and continue until 1 year of age.

Basic characteristics, such as maternal age, prenatal control, use of prenatal steroids, history of maternal chorioamnionitis, diabetes mellitus, preeclampsia, premature rupture of membranes, mode of delivery, gestational age at birth, neonatal sex and weight, growth restriction, respiratory distress syndrome, Apgar score at 1 and 5 min, mechanical ventilation, CPAP nasal, oxygen in nasal cannulas, and intraventricular hemorrhage, were collected at the time of birth by a neonatologist.

### Primary End Point

The primary outcome was to compare the serum Ig concentration (IgG, IgM, and IgA) after the OPAC in the first 4 days of life in premature newborns ≤32 weeks of gestation and to evaluate whether OPAC increases the immunity of premature infants with a gestational age of ≤32 weeks (WG). The serum concentrations of Igs, namely, IgG, IgM, and IgA, at baseline, on day 7, and at 28 days of life, were compared between groups.

### Secondary Outcomes

The secondary outcomes include the following neonatal morbidity: frequency of neonatal sepsis, NEC, BPD, intraventricular hemorrhage, retinopathy of prematurity (ROP), and neonatal death at 28 days, the days to star and complete the enteral feeding, oxygen administration, and compare the total days of hospitalization in both groups.

### Definition of Variables

Feeding With Whole Human Milk

Newborn fed with milk from its mother and/or pasteurized donated milk.

#### Mixed Feeding

It is the feeding with human milk and requires completing the milk intake with any other substitute. Diagnosis of food intolerance was defined as the inability to digest enteral feeding associated with increased gastric residuals, vomiting, abdominal distention, visible bowel loops, diarrhea, or bloody stool (15, 16). The diagnosis of sepsis was made based on the modified Haque KN clinical (17) and hematological criteria. Sepsis: A positive blood culture of PCR in the presence of clinical signs and symptoms of infection (18). The diagnosis of NEC was made using the Bell classification, modified by Walsh and Kliegman (19). Episodes of suspected sepsis were those in which the neonate had at least two clinical or laboratory signs suggestive of infection, along with a negative blood culture result. For the diagnosis of BPD, the 2005 consensus definition was used (20). The definition of ROP was made concerning the definition of the International Committee for the Classification of Retinopathy of Prematurity (21). The total days of hospitalization cover the days from birth to discharge from the hospital.

### IgG, IgM, and IgA Immunoglobulins Quantification

Blood samples were extracted at basal, 7 days, and 28 days after administration of placebo or colostrum. The blood was centrifuged, and 50 to 100 μl of serum was separated. The determinations were performed by nephelometric methods using the MININEPH equipment (Binding Site) kits and IgG, IgM, and IgA from the same company. Dilutions varied according to the concentration of the analyte under study in each sample and were made according to the manufacturer's protocol. The concentrations are automatically calculated from a calibration curve stored in the equipment software. The quantity is determined by the equipment automatically.

The sample size was determined using the formula to compare two means using the EPI-INFO version 7 program for windows. The IgA levels determined by Salazar (22) in healthy newborns were taken as a reference, where they report an average serum IgA concentration of 0.051 with a standard deviation of 0.028 g/L; therefore, we expect that, with the administration of colostrum, the serum IgA concentration was increased by 0.025 g/L considering the following parameters with α = 0.01, a power of 90%, β = 0.10, namely, Δ = 0.026, a mean of IgA = 0.051, and standard deviation = 0.028, requiring 35 participants per group.

## Results

**Figure 1:**
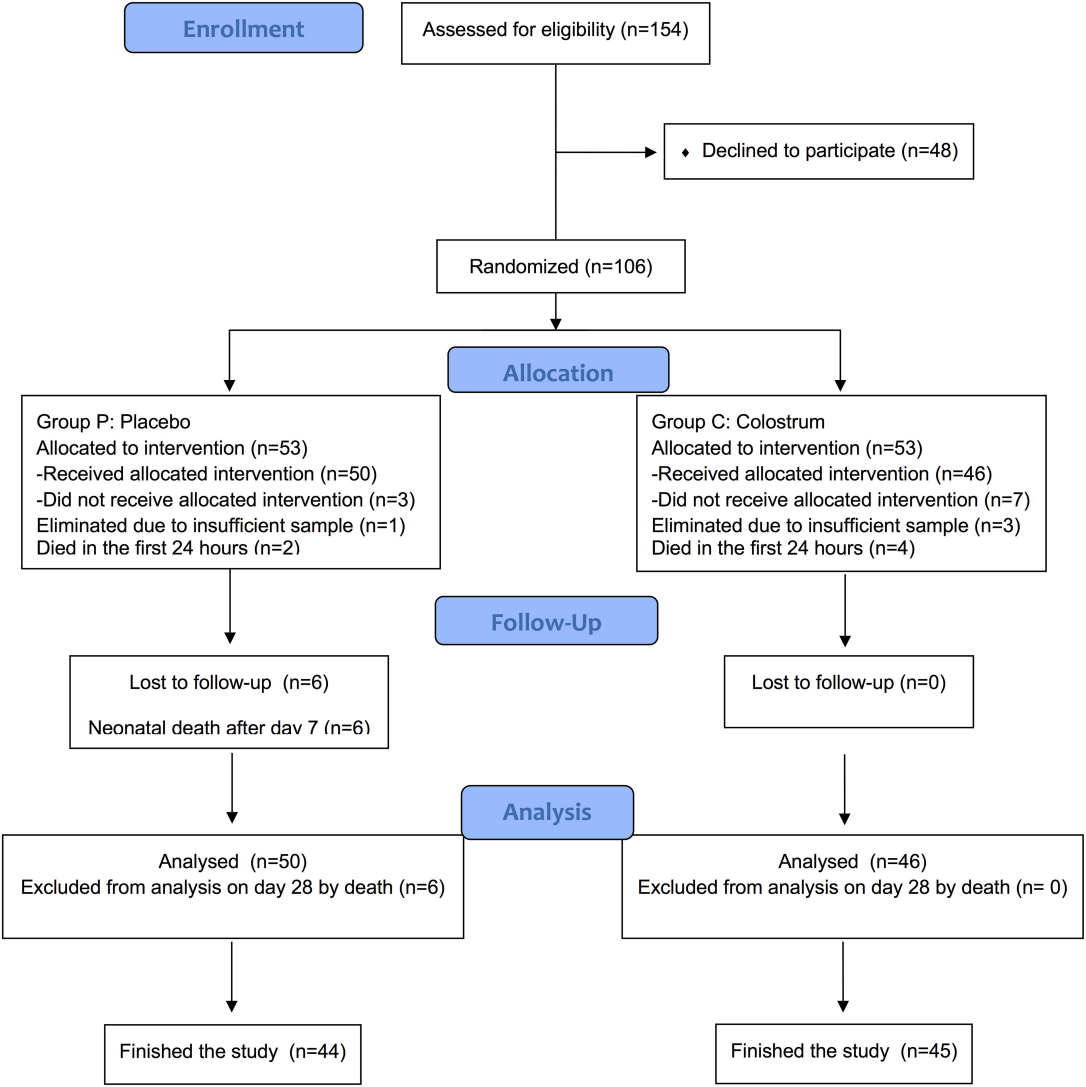
Flow diagram for participants included in the study.

The eligible patients were 154, of which 106 patients were included and randomly assigned, i.e., 53 patients per group. In total, ten participants were eliminated; three participants were eliminated in the placebo group (one by insufficient sample and two died of early sepsis in the first 24 h after entering the study), and seven participants were eliminated in the colostrum group (three by insufficient sample and four died of early sepsis). The remaining 89 participants finished the trial (Figure 1).

The OPAC in premature newborns ≤32 weeks of gestational age was well-tolerated without generating adverse effects.

### Description of the Sample

The age of the mothers of the newborns was similar in both groups; there was no difference in prenatal control, chorioamnionitis, maternal pathology, or the use of prenatal steroids (Table 1).

**Table 1:** Description of the population who completed the treatment.

	**Placebo** ***n* = 50**	**Colostrum** ***n* = 46**	***P*-value**
Maternal age	30.5 ± 6.8	30.1 ± 6.5	0.6
Prenatal control	46 (92%)	39 (84.8%)	0.3
Prenatal steroid	40 (80%)	32 (39.6%)	0.3
Chorioamnionitis	10 (20.4%)	12 (26.1%)	0.4
Diabetes mellitus	10 (20%)	6 (13%)	0.3
Preeclampsia	12 (24%)	15 (32.6%)	0.3
Premature rupture of membranes	18 (36%)	9 (19.5%)	0.06
Vaginal delivery Cesarean section	7 (14%) 43 (86%)	6 (13%) 40 (87%)	0.8 0.8
Respiratory distress syndrome	32 (66.7%)	28 (65.1%)	0.8
1 min Apgar median rank 5 min Apgar median rank	6 1–8 9 3–9	6 1–8 9 2–9	0.8 0.7

*Data are presented as mean ± standard deviation, and frequency (%)*.

Regarding premature newborns, there was no difference between the groups at the time of entry to the study in terms of gender, weight, gestational age, or any other demographic variable (Table 2).

**Table 2:** Description of the population that completed the follow-up.

	**Placebo** ***n* = 50**	**Colostrum** ***n* = 46**	***P*-value**
Gestational age	29.9 ± 1.8	29.3 ± 2	0.1
Female Male	21 (42%) 29 (58%)	23 (50%) 23 (50%)	0.4
Weight	1,111 ± 280	1,147 ± 274	0.9
Start of the enteral administration	2.62 ± 6.8	2.35 ± 9.2	0.9
Total days with supplementary oxygen	45.23 ± 33.5	44.2 ± 33.1	0.8
Days of mechanical ventilation	8.52 ± 15.3	6.6 ± 13.4	0.6
CPAP nasal (Days)	22.1 ± 20.7	19.8 ± 18	0.5
Oxygen in nasal cannulas	15.7 ± 15	17.3 ± 15.6	0.5
Retinopathy of prematurity Stage 1: Stage 2: Stage 3: Stage 4: Stage 5:	12 (24%) 9 (18%) 1 (2%) 0 (0%) 0 (0%) 2 (4%)	14 (30%) 12 (26%) 1 (2%) 1 (2%) 0 (0%) 0 (0%)	0.5 0.3 0.9 0.2 0 0.1
Apnea	30 (60%)	24 (52%)	0.2
Positive blood culture	6 (12%)	4 (8 %)	0.4
Late neonatal sepsis	14 (28%)	16 (34%)	0.7
Days of life at late-onset neonatal sepsis	13.1 ± 14.3	15.7 ± 19	0.6
Neonatal death at 28 days	6 (12%)	0 (0%)	0.06
Intraventricular hemorrhage Grade 1: Grade 2: Grade 3:	16 (32%) 13 2 1	14 (30%) 11 1 2	0.8 0.5 0.5 0.5
Necrotizing enterocolitis stadium >2	7 (14%)	4 (8.6%)	0.3
Patent ductus arteriosus	22 (44%)	13 (28%)	0.04
Intrauterine growth restriction	5 (10%)	4 (8%)	0.8
Bronchopulmonary dysplasia	27 (68%)	26 (67%)	0.5
Days to reach full enteral feeding (days)	17.4 ± 8.4	13.9 ± 7	0.04
Days to reach the birth weight	12.9 ± 4	10.9 ± 2.8	0.01
Length of hospital stay	77.2 ± 47.3	60.2 ± 33.8	0.04

*Data are presented as mean and standard deviation, and frequency (%)*.

### Morbidity Assessment

The time of starting the intervention (placebo or colostrum) was a mean of 18 h after birth (range 10–20 h). The time to the started enteral route was similar in both groups. There was no difference between the groups in the presence of NEC, nosocomial sepsis, retinopathy, BPD, and intraventricular hemorrhage (Table 2).

Regarding other variables, there was a lower number of days in the colostrum group than in the placebo group to complete the enteral feeding 13.9 ± 7 vs. 17.4 ± 8.4 (*p* = 0.04) and reach the birth weight 10.9 ± 2.8 vs. 12.9 ± 4 (*p* = 0.01) and length of hospital stay 60.2 ± 33.8 vs. 77.2 ± 47.3 (*p* = 0.04).

### Serum Immunoglobulin Concentration

The concentrations of Igs at basal, day 7, and day 28 are shown in Table 3. There were no significant differences between groups in the concentrations of IgG and IgM at basal and day 7. The basal concentration of IgA was significantly higher in the placebo group than in the colostrum group (*p* < 0.001); however, the concentration of IgA at day 28 was significantly higher in the colostrum group than in the placebo group (*p* < 0.001). There were no significant differences between groups in the concentration of IgA at day 7.

**Table 3:** Immunoglobulins between groups.

	**Placebo** ***n* = 50**	**Colostrum** ***n* = 46**	***P*-value**
Basal IgG IgM IgA	390 [300–604] 11 [11–23] 16 [5–24]	381 [285–513] 15 [9–22] 5 [3–12]	0.44 0.90 **0.001**
7 days IgG IgM IgA	358 [273–551] 22 [12–33] 16 [10–31]	408 [328–596] 25 [18–35] 19 [11–33]	0.16 0.46 0.10
28 days IgG IgM IgA	262 [169–419] 28 [18–35] 11 [8–18]	272 [159–399] 24 [17–28] 25 [12–35]	0.89 0.06 **0.001**

*Data are presented as median and interquartile. Values with a statistically significant difference are presented in bold*.

**Figure 2:**
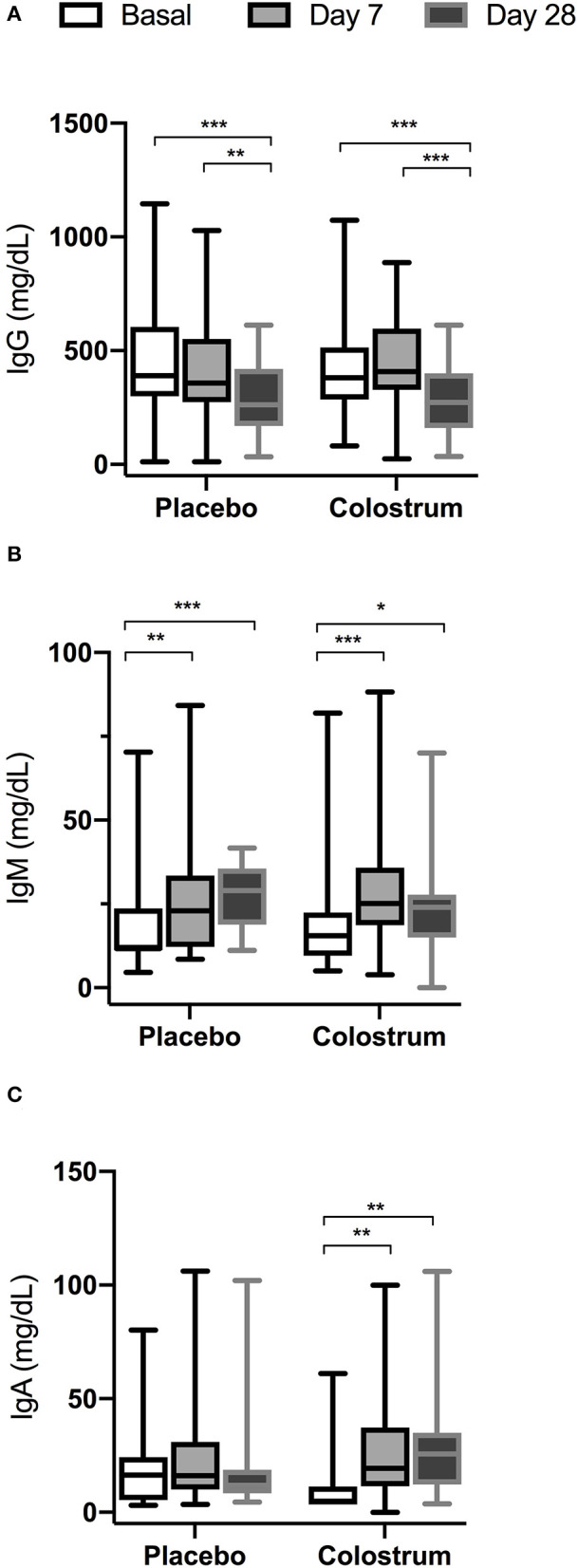
Intragroup comparison of serum immunoglobulin levels at basal, day 7, and day 28. *p < 0.05, **p < 0.001, ***p < 0.0001. **(A)** There was a significant decrease in the intragroup concentration of IgG from basal to day 28 in both groups. **(B)** There was a significant increase in the intragroup concentration of IgM from basal to day 28 in both groups. **(C)** There was a significant increase in the intragroup concentration of IgA from basal to days 7 and 28, respectively, in the colostrum group, while in the placebo group, there was no significant decrease of IgA from basal to day 28.

Intragroup comparison of serum Ig levels is shown in Figure 2 (A,B,C). (A) There was a significant decrease in the intragroup concentration of IgG from basal to day 28 in both groups. (B) There was a significant increase in the intragroup concentration of IgM from basal to day 28 in both groups. (C) There was a significant increase in the intragroup concentration of IgA for 3.8 and 5 times from basal to days 7 and 28, respectively, in the colostrum group, while in the placebo group, there was no significant decrease in IgA from basal to day 28.

### Mortality Analysis

Neonatal mortality at 28 days was lower in the colostrum group vs. placebo group (0 vs. 12%); however, it did not reach a significant statistical difference (*p* = 0.06). The causes of neonatal death are described in Table 4.

**Table 4:** Patients who participated at the study and causes of death.

**Placebo** **cause**	**Colostrum** **cause**
Sepsis shock by *Staphylococcus Aureus*. Died on day 18 of life	
Early-onset neonatal sepsis Congenital pneumonia Necrotizing enterocolitis stage 2-B Died on day 8 of life	
Neonatal sepsis caused by gram negative bacilli Died on day 26 of life	
Late-onset sepsis	
Late-onset sepsis by *Klebsiella* Necrotizing enterocolitis Died on 28 days of life	
Sepsis by *E. Coli* Patent ductus arteriosus Died on day 19 of life	

## Discussion

In this study, the OPAC in premature newborns ≤32 weeks of gestation increased the serum IgA concentration at day 28 and decreased the days to complete enteral feeding, reaching the birth weight and the total days of hospitalization. Interestingly, the basal IgA concentration was higher in the placebo group than in the colostrum group, and it could be attributable to the high proportion of women with premature rupture of membranes in the placebo group 36 vs. 19.6% in the colostrum group, as previously reported by Ismail MA et al. (23).

The increase in serum IgA in the OPAC group compared to the control group demonstrates a positive impact using colostrum, an effect that if it is accompanied by feeding with the mother's milk (homologous), the results at 28 days are improved.

The beneficial effects of OPAC were evidenced as fewer days to reach the enteral route, which suggests the beneficial effect of secretory IgA present in colostrum. These results agree with Palmeira et al. (24) who reported that IgA from human milk constitutes 80–90% of the total proteins present in milk, corresponding to 0.3 g/kg/day. However, only 10% of it is absorbed in the intestine and is transported to the bloodstream, suggesting that the effect of IgA is more specific in the mucosa, favoring tolerance to the enteral route. We must consider that the most significant effect of IgA is the secretory portion, whose action is on the mucosa, and this effect is very difficult to measure. For example, if the quantification of IgA is performed in urine samples as Rodríguez et al. (11) did, only the immunological action on the urinary tract can be measured.

In this study, OPAC was administered with a syringe, which provides a higher concentration of Igs, as reported by Miller J et al. (25). These authors compared the administration of colostrum with a swab vs. the administration with a syringe, and they report that patients who were administered colostrum with a syringe had a higher urinary concentration of IgA and lactoferrin than those administered with a swab. We cannot compare the concentrations of Igs detected with those reported by Miller J et al. (25) since the measurement was made in serum and not in urine.

In this study, there was no statistically significant difference in IgG concentrations between groups, and the concentrations were similar to concentrations reported by Twisselmann N et al. (26) in neonates of 29.8 ± 1.6 weeks of gestation. In this study, IgG levels showed a decrease as age increases in both groups, comparing basal and day 28 of life levels, as reported by Conway et al. (27). The decline in their levels reflects the maternal immune response change, because it is a strictly transferable biomarker (28).

IgM plays a vital role in protecting the mucosa surface of neonates; the human fetus can produce IgM in small amounts and is the one-off Ig usually synthesized by neonates (29, 30), as demonstrated by the identification of IgM-secreting cells in the infant's intestine (31). With respect to IgM, no differences were found between the groups in the three measurements, probably because the colostrum treatment was for a short time, and its effect does not manifest beyond a week, in contrast to that reported by Moreno-Fernandez J et al. (28), in a group of Spanish neonates <32 WG administered 0.2 ml colostrum for every 4 h during 15 days and showed a significant increase of IgM in the colostrum group compared with a control group (28).

Therefore, with the findings of our study, we can suggest that colostrum, in premature newborns, can help improve immunity, and secondary, it can improve tolerance to the oral route.

Regarding the favorable clinical effects that were found, the patients in the group treated with colostrum completed the enteral route earlier, and regained weight in less time, with a statistically significant difference, results similar to those found by Bashir T et al. (32).

We found a decrease in the number of days to reach complete enteral feeding, which is consistent with the findings reported by Garofalo et al. (12), Abd-Elgawad M et al. (13), and Moreno-Fernandez J et al. (28). It was of importance due to early-oral feeding with breast milk having a beneficial impact on neuro-psychomotor outcomes of preterm infants (13). Previous research has shown that the practice of applying mothers' colostrum or milk to the tongue and oropharyngeal pouch of preterm infants prior to gavage feeding results in better feeding tolerance and earlier achievement of enteral and oral feeding (13, 14).

Research similar to our study showed that oral stimulation led to earlier better oral feeding performance, attainment of complete oral feeding, greater weight gain, and a shorter length of hospital stay (33, 34).

Similarly, in this study, the length of hospital stay was significantly lower in the colostrum group, similar to that reported in a recent systematic review (35). It could have to decrease the economic burden of preterm infant care. In a recent study, Rios JD et al. (36) estimated in Canadian dollars a median (IQR) cost of hospitalization before NICU discharge at $30,572 ($16,597–$51,857) at a gestational age of 29–32 weeks; and $100,440 ($56,858–$1,593,867) at a gestational age of <29 weeks, and the cost estimates correlated with length of stay (*r* = 0.97) and gestational age (*r* = −0.65).

No reduction in neonatal sepsis or NEC was found; results comparable with the meta-analysis carried out by Tao J. et al. (35), where they report an RR of 0.59 (033–1.06) for enterocolitis and an RR of 0.78 (0.60–1.03) to reduce sepsis. In this study, the effects of colostrum on neonatal morbidity were similar to the placebo group, considering that the patients were fed in a very similar manner with pasteurized donated and own mother's milk. It is known that patients fed exclusively with human milk have lower rates of NEC compared to formula-fed infants, as described in the systematic review by Miller J et al. (25). With this in mind, we can suggest that colostrum therapy is short but well-tolerated and that we could recommend that mothers extract the colostrum and administer it as early as possible drop by drop on the oral mucosa so that the immunological components can be absorbed. If the patient is fasting, it should be frozen and used when the newborn is ready to receive it.

Regarding mortality in the colostrum group, there were no deaths during the study, showing a tendency to reduce the death of premature newborns in the first month of life compared to the control group where six participants died due to sepsis, which makes us suggest that colostrum has a vital role in modulating the infectious processes of premature newborns; however, when determining the risk reduction, we found no significant changes, data similar to those reported by Rodríguez et al. (11) who also did not find a decrease in neonatal mortality; however, they did find an increase in urinary IgA and lactoferrin, suggestive of an improvement in immunity during the first days of life. Ware JL et al. (37), in a retrospective epidemiological study, found that the earlier the onset of human milk intake, the more statistically significant reduction in mortality (odds ratio [OR] = 0.81, 95% CI 0.68–0.97) (37). Our study did not observe a decrease in NEC, similar to data reported in a recent systematic review (14). One of the patients in the colostrum group died late after 88 days due to severe respiratory distress secondary to severe BPD.

The advantages of this study were that all patients were treated in the same way, which reduces the variability of the results. Colostrum is a free therapy that can be used routinely in all neonatal care units.

The limitations of the study are associated with having it carried out in a single institution, and the sample size was limited for analyzing neonatal mortality.

Future multicentric studies with a large sample size are necessary to corroborate our findings and explore the effect of colostrum administrated during more days.

## Conclusion

These results suggest that the administration of colostrum is safe, considering that we can enhance its effect if we administer it during the period of colostrum production and later continue with the feeding of exclusive human milk due to the potential benefits of intolerance to the enteral route, earlier weight gain. The reduction in the days of hospitalization in the group treated with colostrum shows the relevance of its early application to premature newborns.

## Data Availability Statement

The raw data supporting the conclusions of this article will be made available by the authors, without undue reservation.

## Ethics Statement

The studies involving human participants were reviewed and approved by Internal Review Board of the National Institute of Perinatology Isidro Espinosa de los Reyes. Written informed consent to participate in this study was provided by the participants' legal guardian/next of kin.

## Author Contributions

SR-M: conceptualization. EC-M and EM-O: methodology. DS-B: validation. SR-M, GC, and ES-C: formal analysis. AM-E and MG-C: investigation. SR-M, AM-E, and ER-M: writing-original draft, writing-review and editing. PG-M and SC-M: supervision. JC-P and SR-M: project administration. All authors contributed to the article and approved the submitted version.

## Funding

This research was funded by the Instituto Nacional de Perinatología, Isidro Espinosa de los Reyes, grant number (546) 212250-2320-10305-01-16.

## Conflict of Interest

The authors declare that the research was conducted in the absence of any commercial or financial relationships that could be construed as a potential conflict of interest.

## Publisher's Note

All claims expressed in this article are solely those of the authors and do not necessarily represent those of their affiliated organizations, or those of the publisher, the editors and the reviewers. Any product that may be evaluated in this article, or claim that may be made by its manufacturer, is not guaranteed or endorsed by the publisher.
